# Molecular Dynamics Simulation of Nano-Aluminum: A Review on Oxidation, Structure Regulation, and Energetic Applications

**DOI:** 10.3390/nano16010074

**Published:** 2026-01-05

**Authors:** Dihua Ouyang, Xin Chen, Qiantao Zhang, Chunpei Yu, He Cheng, Weiqiang Pang, Jieshan Qiu

**Affiliations:** 1 School of Chemistry and Chemical Engineering, Nanjing University of Science and Technology, Nanjing 210094, China; 2 Center for Chemical Material Analysis, Nanjing University of Science and Technology, Nanjing 210094, China; 3 Xi’an Modern Chemistry Research Institute, Xi’an 710065, China; 4 School of Chemical Engineering, Beijing University of Chemical Technology, Beijing 100029, China

**Keywords:** nano-aluminum powder, molecular dynamics simulation, ReaxFF reactive force field, energetic materials, nano-aluminum core–shell structure

## Abstract

Nano-aluminum (nAl), characterized by its high combustion enthalpy and enhanced reactivity, serves as a critical component in advanced energetic materials like solid propellants and micro-ignition devices. However, the atomic-scale mechanisms governing its core–shell structure evolution, oxidation dynamics, and interfacial interactions remain elusive to experimental probes due to spatiotemporal limitations. Molecular dynamics (MD) simulations, particularly the synergistic use of a ReaxFF reactive force field (for large-scale systems) and ab initio MD (for electronic-level accuracy), have emerged as a powerful tool to overcome this barrier. This review systematically delineates the oxidation mechanisms and core–shell structure regulation of nAl, with a focus on the multi-scale simulation paradigm integrating DFT, AIMD, and ReaxFF MD that directly supports nAl research. It critically examines the pivotal role of MD simulations in guiding the surface modification of nAl, elucidating combustion mechanisms at the atomic level, and designing interfaces in energetic composite systems. By synthesizing recent advances (2022–2025), this study establishes a clear structure–property relationship between microscopic features and macroscopic performance of nAl. Furthermore, it identifies prevailing challenges, including simulations under multi-physics loading, multi-scale bridging, and quantitative experiment-simulation validation that specifically affect nAl-based energetic systems. Finally, future research directions are prospected, encompassing the development of machine learning-empowered force fields tailored for nAl systems, multi-scale and multi-field coupling simulation frameworks targeting nAl applications, and closed-loop experiment-simulation systems for nAl-based energetic materials. This review aims to provide fundamental insights and a technical framework for the rational design and engineering application of nAl-based energetic materials in fields such as aerospace propulsion.

## 1. Introduction

Since the Lawrence Livermore National Laboratory (LLNL) in the United States introduced nano-aluminum powder (nAl) into the energetic material system in 1995, its high reactivity derived from the nano-effect has increased the combustion rate of thermite by three orders of magnitude and significantly improved energy release efficiency, which has triggered an upsurge in the research of new energetic materials [1]. NAl usually exists in an “Al core-Al_2_O_3_ shell” structure, and shell thickness (2–5 nm) directly determines the active aluminum content and reaction characteristics [2]. However, experimental methods are difficult to capture the atomic-level dynamic process of shell growth [3]. In addition, in the energetic composite system, the microscopic mechanisms such as the interfacial reaction between nAl and oxidants (e.g., CuO, Fe_2_O_3_, etc.) and atomic diffusion paths are the key to regulating ignition delay and energy release efficiency [4], while the transient process under high temperature and high pressure is beyond the scope of traditional experimental characterization [5].

Notably, the research on nAl’s structure regulation and reaction mechanism has substantial practical application potential across multiple high-tech industries: In aerospace propulsion, optimizing nAl’s core–shell structure and oxidation behavior can enhance the specific impulse of solid propellants by 10–15%, enabling longer-range and more efficient rockets and satellites [6]. In micro-ignition devices for automotive airbags and micro-electromechanical systems (MEMSs), precise control of nAl’s ignition delay (targeting 1–5 ms) can improve the reliability and response speed of initiation systems [7]. In military energetic materials (e.g., high-explosive formulations, pyrotechnic compositions), improving nAl’s energy release efficiency can increase the detonation pressure and blast power of warheads while reducing their overall mass [8]. In civil engineering, nAl-based thermite composites with tailored reaction rates are being developed for on-site welding of large-scale steel structures and emergency repair of infrastructure [9]. These industrial applications urgently require atomic-level insights into nAl’s behavior, which molecular dynamics simulations are uniquely positioned to provide.

The breakthrough of molecular dynamics simulation technology provides a new approach to solve the above nAl-specific problems: Density Functional Theory (DFT) can accurately calculate the interfacial electronic structure of nAl–oxidant systems as the theoretical basis [10], ab initio MD (AIMD) can capture the electronic dynamic behavior of initial chemical reactions involving nAl [11], and ReaxFF MD realizes the nanosecond-scale reaction simulation of ten-million-atom nAl-containing systems to make up for the spatiotemporal limitation of AIMD [12]. These three methods form a synergistic technical system covering from electronic structure to large-system dynamic evolution for nAl research. Cui et al. [4] pointed out in their review that the synergistic application of the three simulation methods has become a core tool for analyzing the reaction mechanism of nano-thermite. To ensure the rigor and reproducibility of this review, the relevant literature (2022–2025) was systematically retrieved from Web of Science, Scopus, CNKI, and Google Scholar using keywords including “nano-aluminum”, “molecular dynamics simulation”, “ReaxFF”, and “energetic materials”, with studies selected based on their focus on nAl-based systems, the employment of MD simulation as a core tool, and the provision of quantitative data or clear mechanism insights. It can be concluded that existing studies are mostly limited to a single heating condition, and the simulation coverage of nAl-based energetic composite systems is still inadequate. On this basis, based on the logical thread of “structural characteristics–simulation analysis–application design”, this paper mainly integrates the research results of the past three years, focuses on highlighting the application value of MD simulation in the field of nAl-containing energetic materials, and provides theoretical references for the directional optimization of nAl ‘s structure, reactivity, and composite system compatibility.

## 2. Structural Characteristics and Oxidation Behavior of nAl

### 2.1. Core–Shell Structure and Its Impact on Reactions of nAl

In a nano-energetic material system, the “core–shell structure” of nAl is the core feature that fundamentally distinguishes it from micro-aluminum powder. Among them, the thickness parameter and crystal structure characteristics of the shell layer have a significant regulatory effect on the reactivity of nAl, and this correlation is a key entry point for understanding its energy release behavior.

#### 2.1.1. Shell Growth Kinetics and Atomic Diffusion Mechanism of nAl

**Figure 1:**
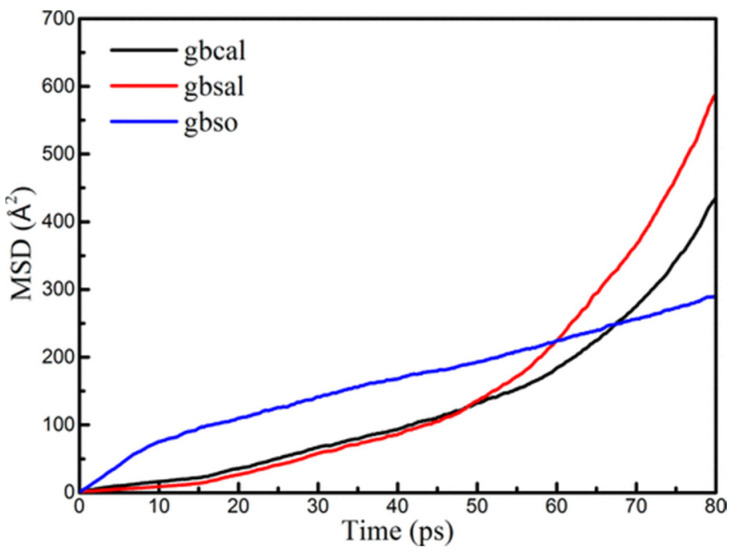
Time evolutions of atomic mean square displacements at the core–shell interfaces [3].

Regarding the dynamic growth kinetics of the shell layer in the core–shell structure of nAl, Rai et al. [13] observed through in situ high-resolution transmission electron microscopy (HRTEM) that when nAl with a particle size of 20–30 nm is exposed to a high-temperature environment above 1000 K, it gradually forms a hollow structure. The microscopic process is manifested as the diffusion of Al atoms from the core to the outer side of the shell, while O atoms from the external environment migrate to the inner side of the core, eventually leading to dynamic changes in the thickness and structure of the shell layer. To further verify this experimental phenomenon and reveal the atomic-scale mechanism, Zeng et al. [3] adopted the ReaxFF reactive force field molecular dynamics (MD) simulation method targeting nAl. This approach not only computationally confirmed the aforementioned diffusion process of Al and O atoms in nAl’s core–shell structure but also drew a key conclusion through quantitative analysis: the mean square displacement (MSD) of O atoms is approximately 2.3 times that of Al atoms (as shown in Figure 1, the legends gbcal/gbsal/gbso refer to Core/Al, Shell/Al, Shell/O). The significant value of this simulation result lies in that it not only verifies the mechanism of the hollow structure formation process observed in experiments but also clarifies that O atoms have higher diffusion activity in the oxide layer of nAl from the perspective of atomic motion. This provides a clear microscopic mechanism explanation for the dynamic evolution process of nAl’s core–shell structure with temperature and time, and also offers a theoretical basis for the subsequent regulation of nAl’s shell growth rate.

#### 2.1.2. Reactivity Regulation Bottleneck and Optimal Size Range of nAl

In the process of regulating reactivity by the core–shell structure of nAl, there exists a key issue of the “reactivity regulation bottleneck”. Studies by Dlott et al. [14] have shown that when the particle size of nAl is less than 30 nm, the volume proportion of the Al_2_O_3_ shell generated on its surface will exceed 20%. An excessively thick shell will form a “diffusion barrier” that hinders the contact between Al atoms and the reaction medium, thereby significantly reducing the reactivity of nAl. This conclusion that “smaller particle size leads to decreased reactivity of nAl” is highly consistent with the results obtained by Aumann et al. [15] through laser ignition experiments on nAl. Both jointly confirm that the reactivity of nAl does not continuously increase with the decrease of particle size, but there exists an “optimal size range”. Specifically, when the particle size of nAl is in the range of 30–50 nm, it can not only avoid the formation of a diffusion barrier due to the excessively high proportion of the shell layer but also give full play to the advantage of the specific surface area at the nano-scale, thereby achieving the optimal reactivity.

However, most of the current simulation studies on the core–shell structure and reactivity are limited to the ideal spherical particle model, which ignores the morphological irregularities (such as quasi-spherical, flaky, chain-like, etc.) and particle size distribution broadening that are unavoidable during actual preparation. These factors will lead to differences in shell thickness distribution, specific surface area, and interfacial contact mode between actual particles and ideal models, resulting in deviations between simulation-predicted reactivity and the experimental results. Up to now, only a few studies have attempted to simulate quasi-spherical particles with simple shape deviations, and there is a lack of systematic simulation analysis on the quantitative impact of different morphologies and particle size distributions on reactivity. This has become one of the urgent directions to break through in the current research on reactivity regulation.

#### 2.1.3. Storage Stability and Surface Adsorption Mechanism of nAl

The stability of the core–shell structure also directly affects the storage performance of nAl, and the attenuation of storage stability is essentially the result of the interaction between the core–shell structure and environmental media. To reveal its microscopic essence, MD simulations by Zeng et al. [3] further pointed out that the adsorption energy of H_2_O molecules on the surface of the Al_2_O_3_ shell is approximately −0.8 eV (a negative adsorption energy indicates that the adsorption process proceeds spontaneously). This strong adsorption effect will drive H_2_O molecules to participate in the oxidation reaction of the shell layer, eventually leading to an increase in shell thickness and the coverage of active sites. The core significance of this study is that it establishes a quantitative correlation between the macroscopic reactivity attenuation rate and the microscopic H_2_O molecule adsorption process for the first time. It provides a clear theoretical guidance direction for inhibiting H_2_O molecule adsorption and improving storage stability through surface modification (such as coating an inert protective layer, introducing hydrophobic groups, etc.).

#### 2.1.4. Application Verification of nAl’s Core–Shell Structure in Thermite Reactions

The aforementioned laws and mechanisms regarding the core–shell structure have been verified in practical energetic material systems. Maini et al. [16] successfully prepared μAl@NiO micro-composite particles with a core–shell structure through a wet chemical method. During the preparation process, the Al core (neutral metallic) and NiO shell (ionic) achieve surface charge modification: the Al core is treated to carry a weak positive charge, while the NiO shell exhibits a weak negative charge due to its surface oxygen-rich groups. This charge distribution enables electrostatic self-assembly—the mutual electrostatic attraction between the oppositely charged surfaces overcomes the inherent repulsion between neutral metal and ionic solids, realizing close contact between the core and shell. Combined with multi-dimensional methods such as simultaneous thermal analysis (TG-DSC), combustion performance testing, and molecular dynamics simulation, they systematically revealed the key regulatory role of the core–shell interface in thermite reactions. The research results show that this interface bonding method not only significantly reduces the initial temperature of the thermite reaction but also greatly improves the energy release rate and combustion efficiency of the reaction. At the same time, MD simulations further verified that the reaction path of this system is dominated by the diffusion mechanism of molten Al atoms into the NiO shell. This diffusion-dominated reaction path can effectively promote the rapid formation of Al-Ni alloy phases (such as AlNi, Al_2_Ni_3_, etc.), thereby achieving efficient energy release. This case not only confirms the optimization effect of the core–shell structure on reaction performance but also provides a reference idea for the structural design of other energetic material systems.

### 2.2. Experimental and Simulative Understanding of nAl’s Oxidation Mechanism

In the field of nano-energetic materials, the oxidation process of nAl is a core link that determines its energy release efficiency, ignition characteristics, and storage stability. This process involves multi-stage and multi-scale atomic motion and reaction behaviors. With the continuous development of computational simulation technology, molecular dynamics (MD) simulation has become an important complementary method to experimental research. The combination of the two has gradually clarified the core mechanism of nAl oxidation and promoted a systematic understanding of this complex process in the academic community.

#### 2.2.1. Diffusion-Oxidation Mechanism of nAl: Experimental Observation and Simulative Refinement

**Figure 2:**
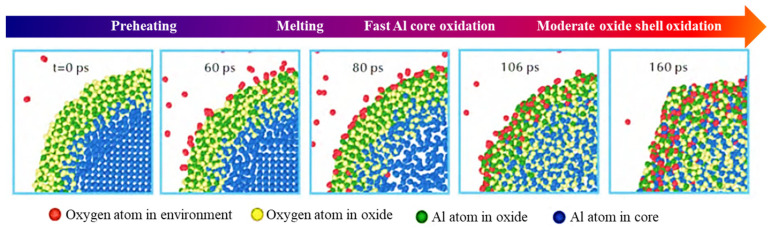
Schematic diagram of the four oxidation stages of ANP [18].

In the research on the oxidation mechanism of nAl, the diffusion-oxidation mechanism is one of the earliest concerned and most in-depth research directions. Based on experimental phenomena, Rai et al. [17] proposed that the core control step in the initial stage of oxidation is the diffusion process of O atoms into the aluminum core, which laid the foundation for subsequent mechanism research. To further refine the dynamic process of this mechanism, Chu et al. [18] used ReaxFF reactive force field molecular dynamics simulation to accurately divide the complete oxidation process of nAl into four consecutive stages: preheating, melting, aluminum core oxidation, and secondary oxidation of the oxide layer (as shown in Figure 2). It is worth noting that the stages divided by this simulation are highly consistent with the distribution characteristics of exothermic peaks in differential scanning calorimetry (DSC) experiments—for example, the preheating stage corresponds to a weak endotherm, and the melting and aluminum core oxidation stages correspond to strong exothermic peaks. This fully reflects the complementarity of a simulation in explaining mechanism details and verifying experimental phenomena, enabling the diffusion-oxidation mechanism to evolve from macroscopic experimental speculation to a clear quantitative description of microscopic processes.

#### 2.2.2. Melting-Dispersion Mechanism of nAl: Mechanism Breakthrough and Quantitative Support Under Extreme Conditions

Regarding the oxidation behavior of nAl under extreme heating conditions, the academic community has proposed the melting-dispersion mechanism to explain special phenomena that cannot be covered by conventional diffusion models. As early as 2007, Levitas et al. [19] pointed out through theoretical analysis that when the heating rate reaches an extremely high level of 10^6^–10^8^ K/s, the Al core of nAl will expand due to rapid heating (a volume increase of about 6%). The internal stress generated by this expansion is sufficient to cause the rupture of the surface Al_2_O_3_ shell, and then the molten Al clusters will scatter at a high speed of 100–250 m/s, and then react rapidly with the surrounding oxidants. This mechanism lacked quantitative parameter support for a long time—until 2021, when Zhong et al. [20] successfully captured the critical pressure threshold (about 2.3 MPa) for shell rupture through ReaxFF MD simulation when studying the behavior of nAl in the high-energy environment of RDX (cyclotrimethylenetrinitramine). This critical pressure directly corresponds to the internal stress generated by the thermal expansion of the Al core as proposed by Levitas: when the heating rate exceeds 10^6^ K/s, the Al core melts and expands, generating internal pressure that exceeds 2.3 MPa, leading to shell rupture and the subsequent scattering of molten Al. Thus, this quantitative result provides direct microscopic evidence for the melting-dispersion mechanism by verifying the key stress threshold required for shell rupture, and clarifies the triggering conditions of this mechanism, providing an important basis for predicting the oxidation behavior of nAl under extreme working conditions (such as an explosion, pulse heating).

#### 2.2.3. Ion Diffusion Mechanism of nAl: Electric Field Effect and Simulation-Experiment Synergistic Verification

In addition to atomic diffusion, the role of ion migration in the oxidation process of nAl has also been gradually revealed, forming the ion diffusion mechanism. Through electrochemical experiments, Henz et al. [21] proposed that the intrinsic electric field existing inside the Al_2_O_3_ shell can significantly accelerate the diffusion process of Al ions, and the migration contribution of Al ions accounts for more than 90% of the total ion mass flux. This discovery subverts the traditional cognition that only focuses on O atom diffusion. To verify the dynamic change law of the electric field effect, Zeng et al. [3] further carried out a ReaxFF MD simulation. The results show that when the temperature rises to 2000 K, the Al_2_O_3_ shell melts, the interface electric field strength drops sharply to 0, and the ion diffusion resistance (originally enhanced by the electric field) disappears completely; this leads to a sudden increase in the diffusion rate of Al ions and O ions, thereby significantly accelerating the oxidation reaction. This simulation result is highly consistent with the phenomenon of “oxidation kinetics mutation at high temperature” in subsequent electrochemical tests [22]: the tests showed that when the temperature exceeds 2000 K, the oxidation rate of nAl increases by an order of magnitude, which matches the simulation’s prediction of ion diffusion resistance disappearance. This cross-scale verification clearly clarifies the regulatory mechanism of temperature on the ion diffusion-dominated oxidation process.

To better distinguish and apply the three oxidation mechanisms in practical research and engineering scenarios, Table 1 summarizes their key trigger conditions, dominant particle size ranges, and typical application scenarios. This table not only systematically sorts out the core characteristics of each mechanism but also provides a clear reference for researchers to select the appropriate mechanism model according to specific working conditions (such as heating rate, pressure, particle size) when studying the oxidation behavior of nano-aluminum powder. For example, in the design of slow-ignition solid propellants, the diffusion-oxidation mechanism is more applicable due to the relatively low heating rate (<10^5^ K/s); while in the research of high-energy explosion devices, the melting-dispersion mechanism should be the focus of attention because of the extreme heating rate (>10^6^ K/s) and high pressure (>2.3 MPa) in the system.

The proposal and gradual verification of the three mechanisms (diffusion-oxidation, melting-dispersion, and ion diffusion) reflect the deepening process of the academic community’s understanding of the nAl oxidation process from a single perspective to multi-mechanism and multi-condition perspective. Among them, MD simulation not only plays an irreplaceable role in explaining mechanism details and obtaining quantitative parameters but also shows unique advantages in identifying the dominant conditions of different mechanisms (such as temperature, pressure, heating rate), becoming a core bridge connecting microscopic atomic behavior and macroscopic oxidation performance of nAl.

#### 2.2.4. Expansion of Related Simulation Studies and Their Reference Significance

**Figure 3:**
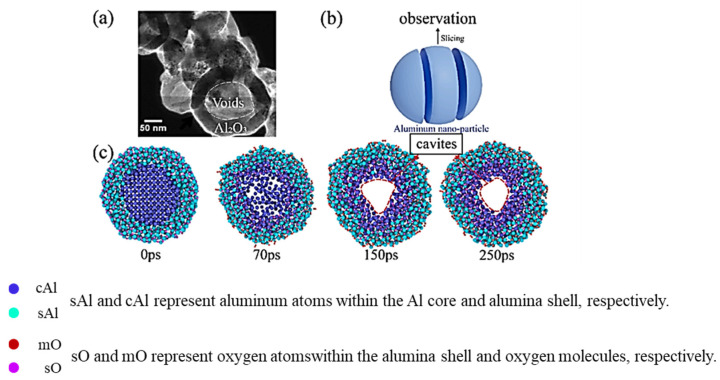
Analysis of cavity evolution during oxidation of aluminum nanoparticles [23].

Han et al. [23] constructed aluminum nanoparticle models with various particle sizes via molecular dynamics simulation and probed their oxidation processes using the reactive force field (ReaxFF). As illustrated in Figure 3, this study investigated the evolution mechanism of internal cavities during the reaction of aluminum nanoparticles with oxygen, and the underlying formation mechanism was clarified as follows: during oxidation, the inward diffusion of O atoms outpaces the outward diffusion of Al atoms, resulting in a dense outer oxide layer; continuous oxidation and consumption of the internal Al core generate voids that gradually aggregate into cavities, with 20 nm identified as the critical particle diameter for cavity formation, particles smaller than this threshold form uniform oxide layers free of cavities, whereas larger particles develop distinct internal cavities owing to such diffusion asymmetry [24,25]; and future research could focus on the synergistic regulation of particle size and surface modification to further enhance the reactive activity and storage stability of nAl for energetic material applications [26,27]. The study finally clarified the intrinsic link between atomic diffusion behavior and cavity evolution. This study emphasizes the influence of particle size on the cavity evolution mechanism and provides new insights into the atomic-scale oxidation mechanism of metal nanoparticles.

In addition to studies directly targeting nAl, simulation studies on the oxidation of other nano-metals and oxidant decomposition also provide important references for deepening the understanding of the nAl oxidation mechanism, further enriching the idea of “experiment-simulation” collaborative research. Although the core research objective of Reference [28] is the photothermal response of aluminum micro-nano particles, using finite element simulation as the main method, the paper systematically cites a number of molecular dynamics simulation results, focusing on revealing the oxidation mechanism of aluminum nanoparticles under rapid heating conditions. Among them, MD simulation clearly shows that the oxidation behavior of aluminum nanoparticles can be subdivided into different modes such as atomic diffusion, shell rupture, and micro-explosion, and even at ultra-high heating rates (>10^12^ K/s), the diffusion process still dominates the oxidation reaction. This conclusion not only fills the knowledge gap in understanding the oxidation mechanism of nAl under extreme heating conditions and provides an atomic-scale perspective for the ignition and combustion mechanism of aluminum particles in energetic materials, but also echoes the aforementioned diffusion-oxidation mechanism, supporting the phenomenon of “diffusion-dominated oxidation coexisting with sputtering” observed in experiments and further verifying the universality of the diffusion mechanism.

Reference [29] focuses on silver nanoparticles and systematically explores the oxidation kinetics and crystal structure evolution law of silver nanoparticles in a high-temperature environment using the ReaxFF reactive force field method. Although the research object is silver, the simulation strategy adopted in this study (such as kinetic tracking under multiple temperature gradients, and quantitative analysis of core–shell structure evolution during oxidation) and the optimization idea of ReaxFF force field parameters have direct reference significance for the oxidation simulation of nAl. Specifically, the method of establishing a quantitative relationship between particle size/temperature and oxidation rate can be directly applied to the study of nAl; the force field parameter optimization strategy for metal–oxygen bond interaction provides a reference for improving the accuracy of an Al-O force field in a nano-aluminum oxidation simulation. Its research findings show that nanoparticle size and temperature have significant regulatory effects on oxidation rate, core–shell structure formation, and crystal order loss. For example, small-sized particles have an oxidation rate 2~3 times that of large-sized particles due to their large specific surface area; increasing the temperature accelerates the disordering of the crystal structure. These laws provide an analog reference for understanding the relationship between “size, temperature, and –oxidation performance” during the oxidation of nAl, and its quantitative analysis method also provides a reusable technical path for the study of nAl oxidation kinetics.

## 3. Molecular Dynamics Simulation Methods for nAl

**Figure 4:**
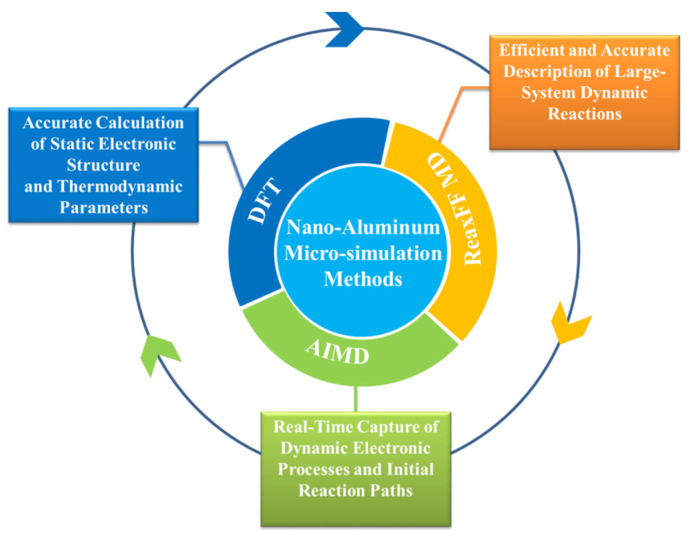
Simulation technical framework for nAl from static properties to dynamic reactions.

In the research on the microscopic mechanisms and performance regulation of nAl, molecular dynamics (MD)-related simulation technologies are core tools for revealing atomic-level behaviors and correlating macroscopic properties. To meet the research needs of nAl systems, a three-level progressive simulation system of “Density Functional Theory (DFT)–Ab Initio Molecular Dynamics (AIMD)–Reactive Force Field Molecular Dynamics (ReaxFF MD)” has been formed, as shown in Figure 4. The DFT provides high-precision electronic structure parameters for small systems (hundreds of atoms, time scale: 0.01 ps); AIMD captures initial dynamic reaction processes with electronic-level resolution (thousands of atoms, time scale: 40 ps); and ReaxFF MD realizes large-system evolution simulation (ten million atoms, time scale: nanosecond level). The three methods form a complete technical framework covering from static properties to dynamic reactions.

### 3.1. Core Simulation Methods and Technical Characteristics of Molecular Dynamics for nAl

#### 3.1.1. DFT Simulation: Accurate Calculation of Static Electronic Structure and Thermodynamic Parameters

As the “accuracy benchmark” in the three-level system, DFT simulation has the core advantage of accurately describing electronic structures and static thermodynamic properties without relying on empirical parameters. It is mainly used for basic theoretical calculations of key interfacial interactions, energy parameters, and reaction paths in nAl systems, and is usually implemented using mature software packages such as VASP and CASTEP [30]. Its technical characteristics determine that this method is more suitable for a static analysis of small systems (usually within hundreds of atoms) rather than tracking dynamic reaction processes.

In the design of nAl energetic composite systems, the DFT simulation provides key theoretical support for oxidant crystal plane selection and interfacial interaction mechanism analysis. For example, Xue et al. [10] studied the interfacial interaction between Al and Fe_2_O_3_ (a commonly used oxidant) through DFT calculations and found that when the Fe_2_O_3_ surface is the (104) crystal plane with exposed O atoms, the interfacial adhesion work with Al reaches 3.8 J/m^2^, which is three times the adhesion work of the surface with exposed Fe atoms. This result clearly shows that the Fe_2_O_3_ crystal plane with exposed O atoms can form a stronger interfacial bond with Al, which can significantly promote atomic diffusion and energy transfer during the reaction process. It provides clear theoretical guidance for the crystal plane regulation and interface optimization of Fe_2_O_3_ oxidants in energetic composite systems and also reflects the irreplaceability of the DFT in the calculation of static key parameters.

#### 3.1.2. AIMD Simulation: Real-Time Capture of Dynamic Electronic Processes and Initial Reaction Paths

AIMD simulation is built based on the DFT theoretical framework. It inherits the high-precision advantage of the DFT in electronic structure description and breaks through the limitation of static calculation. It can update the electronic structure in real-time during atomic motion, thereby capturing dynamic electronic behaviors in chemical reactions (such as chemical bond breaking/formation, charge transfer, etc.). It is a key bridge connecting static electronic properties and dynamic reaction processes. However, its technical characteristics also have obvious limitations: since most common AIMD implementations (e.g., Born-Oppenheimer MD) require solving the electronic Schrödinger equation at each step to obtain the potential energy surface, the calculation cost is extremely high. While the Car-Parinello MD method (an alternative AIMD approach that treats electronic and ionic degrees of freedom simultaneously without explicitly solving the Schrödinger equation at each step) offers computational advantages for certain systems, it is not obsolete and remains widely used for specific applications such as extended systems or long-time-scale dynamics. Nevertheless, regardless of the specific implementation, the AIMD simulation system scale is usually limited to within 1000 atoms and the time scale is mostly at the picosecond (ps) level, making it difficult to cover the long-term evolution process of large systems [31]. Despite the spatiotemporal scale limitations, AIMD still has unique advantages in revealing the initial path and core mechanism of the rapid reaction of nAl. As early as 2009, Shimojo et al. [11] studied the interfacial reaction of an Al/Fe_2_O_3_ thermite system through AIMD simulation and proposed the key mechanism of “metal–oxygen inversion”. The simulation results showed that in the initial stage of the reaction, O atoms would detach from the Fe_2_O_3_ lattice within 0.2 ps and migrate rapidly to the Al side, forming a preliminary structure of Al-O bonds. This dynamic process is highly consistent with the interfacial atomic migration phenomenon observed by subsequent in situ transmission electron microscopy (TEM), providing direct atomic-level evidence for the initial initiation mechanism of the thermite reaction.

With the improvement of computing power, the application of AIMD in the analysis of multiple reaction stages has gradually expanded. In 2021, Feng et al. [32] studied the oxidation reaction of the Al/NiO system through AIMD simulation and confirmed for the first time that the system has the characteristic of a “secondary exotherm”, and clarified that this characteristic corresponds to two different reaction stages: the first stage is the rapid interfacial reaction between Al and NiO (corresponding to the primary exotherm), and the second stage is the deep diffusion reaction of Al atoms into the NiO bulk phase (corresponding to the secondary exotherm). To further verify the reliability of the simulation results, the researchers compared the reaction initiation temperature obtained by AIMD with the test results of different experimental methods (DSC, TG-DTA, laser ignition, high-pressure DSC) (see Table 2). It was found that the deviation between the simulated values and experimental values was within a reasonable range (e.g., the simulated primary exotherm initiation temperature was 729–904 K, and the experimental value was 673–774 K), which fully proves the accuracy of AIMD in dynamic reaction stage division and key temperature parameter prediction.

It is worth noting that although AIMD can accurately capture rapid electronic processes and initial reaction paths, the limitation of spatiotemporal scales makes it difficult to simulate the large-system evolution of nAl in practical applications (such as multi-particle agglomeration, long-term storage oxidation, etc.). This limitation has also prompted researchers to seek connection with ReaxFF MD. Through the combination of “small-system accurate mechanism (AIMD)—large-system dynamic evolution (ReaxFF MD)”, complete simulation coverage from mechanism to application is achieved.

#### 3.1.3. ReaxFF MD Simulation: Efficient and Accurate Description of Large-System Dynamic Reactions of nAl

ReaxFF MD simulation describes the chemical reaction process of nAl-containing systems through the core parameter of “bond order” (bond order changes with atomic distance and can reflect the breaking and formation of chemical bonds in real-time). It achieves a good balance between calculation accuracy and simulation efficiency for nAl systems; it not only avoids the high calculation cost of DFT/AIMD for large nAl systems but also breaks through the limitation that traditional non-reactive force fields cannot describe chemical reactions involving nAl. It can realize the dynamic simulation of nAl-containing systems with thousands to millions of atoms and nanosecond (ns)-level time scales [33]. Notably, ReaxFF is not the only force field for large-scale MD simulations of nAl systems; other force fields such as tight-binding potentials (e.g., those implemented in DFTB+) and embedded atom method (EAM) potentials also have their applicable scenarios and cannot be omitted.

The prevalence of ReaxFF in the cited studies is justified by its unique advantages tailored for nAl-based energetic systems, despite its initial design for organic/covalent materials: First, ReaxFF force fields have been continuously optimized and parameterized for metal–oxide systems (e.g., Al/Al_2_O_3_) in recent years, with dedicated adjustments to Al-O bond interactions, charge transfer models, and metal–oxide interface behaviors [34,35]. These optimizations enable an accurate description of chemical reactions involving Al and its oxides, such as shell growth, oxidation, and interfacial bonding with oxidants. Second, compared to EAM potentials (which focus on metallic bonding and are primarily suitable for non-reactive processes like sintering or mechanical deformation of nAl), ReaxFF can directly simulate bond breaking/formation during oxidation and thermite reactions—critical for capturing the core dynamic behaviors of nAl in energetic materials. Third, relative to tight-binding potentials like DFTB+, ReaxFF maintains lower computational costs while achieving sufficient accuracy for large-system simulations (e.g., ten-million-atom scale), making it feasible to track long-time-scale processes such as multi-particle agglomeration, oxide layer evolution, and bulk thermite reactions.

Tight-binding potentials (e.g., DFTB+) are valuable supplements for large-scale simulations of nAl systems, as they balance quantum mechanical accuracy and computational efficiency by approximating electronic structure calculations. They are particularly suitable for scenarios requiring a semi-quantitative description of electronic effects (e.g., charge transfer at nAl–oxidant interfaces, band structure changes during oxidation) in medium-sized systems (10^4^~10^5^ atoms) [36]. For example, DFTB+ has been used to study the initial oxidation stage of nAl by capturing the charge redistribution between Al and O atoms, providing insights into the electronic driving force of oxidation [37]. EAM potentials, on the other hand, are widely used for non-reactive simulations of nAl, such as predicting the mechanical properties of nAl particles, sintering behavior without chemical reactions, or morphological changes under pressure [38]. However, their inability to describe bond breaking/formation limits their application in reactive processes like nAl oxidation and thermite reactions.

The reliability of ReaxFF MD in nAl simulation depends on the continuous optimization and experimental verification of force field parameters. In 2012, Song et al. [34] compared the energy release data of nAl measured by ReaxFF simulation and experiments and confirmed that the description error of this force field on the Al energy release process was less than 8%, which initially verified its accuracy in energy property simulation. With the in-depth research, Reference [35] optimized and developed the ReaxFF-C/H/O/Al/F force field to meet the simulation needs of the C/H/O/Al/F system. Compared with traditional Al-O force fields, this optimized force field adds precise interaction parameters for F-Al bonds and modifies the bond energy parameters of C-F and Al-O bonds in the high-temperature region (1200–2000 K), effectively solving the problem of inaccurate reaction path prediction in cross-temperature region simulation. The calculation results of key properties such as PVDF crystal structure (lattice parameter error < 3%), lattice parameters, and dipole moment are highly consistent with quantum mechanics calculations and experimental data, which greatly improves the description accuracy of PVDF-condensed phase properties and Al/PVDF reaction processes. More importantly, the optimized force field has good transferability for both non-reactive conformational transitions in the low-temperature region and chemical transformations in the high-temperature region. It can stably simulate the reaction kinetics between PVDF and surface-oxidized nano-aluminum at 1200–2000 K: the simulated ignition delay time of the Al/PVDF system is 12.3 ns, which is consistent with the experimental result of 11.8 ns (error < 4%); and the predicted main reaction products (AlF_3_, CO_2_) are also consistent with mass spectrometry test results. This solves the parameter inaccuracy problem that traditional force fields are prone to in cross-temperature region simulation, provides a reliable simulation tool for research on the reaction mechanism of nAl and PVDF-based energetic composite materials under extreme working conditions (such as rocket propellant combustion), further expands the application scope of ReaxFF MD in nAl composite systems, and realizes an efficient and accurate description of large-system dynamic reactions.

### 3.2. Force Field Development and Latest Progress in Molecular Dynamics Simulation of NA

In the molecular dynamics simulation research of nAl, a force field serves as the core bridge connecting microscopic atomic interactions and macroscopic simulation results. Its applicability and accuracy directly determine the reliability of simulation results, and it is a key prerequisite for ensuring that simulations can effectively guide experimental research and material design. With the increasing application demands of nAl in multi-component energetic systems and extreme working conditions (high temperature, high pressure, impact load), during the period of 2022–2025, the research focus of force field development has gradually concentrated on three directions: “adaptability optimization for multi-component systems”, “parameter calibration under extreme conditions”, and “specialized design for energetic composite systems”. A series of breakthrough research results have been achieved, significantly expanding the application boundary of molecular dynamics simulation in the field of nAl. In addition to ReaxFF, the development of tight-binding potentials (e.g., DFTB+) and EAM potentials for nAl systems has also made progress: tight-binding potentials have been optimized for Al-O-Cu/Ni multi-component interactions, while EAM potentials have been calibrated for the high-temperature mechanical properties of nAl, enriching the force field toolkit for nAl simulation.

#### 3.2.1. Multi-Component Force Fields: Cross-System Adaptability and Machine Learning Empowerment

In view of the characteristic that nAl is often compounded with various oxidants (such as NiO, CuO, etc.) to form energetic systems, the development of multi-component force fields aims to solve the problem of the limited applicability of force fields for single systems and realize an accurate description of reaction behaviors in different composite systems. In 2021, Li et al. [39] took the lead in developing specialized ReaxFF-AlNiO force field parameters for an Al/NiO composite system. By supplementing experimental and DFT data on the interactions of Al-Ni and Ni-O bonds, the simulation error of the reaction heat of this system was reduced from 15% of the traditional general force field to 9%, providing more reliable force field support for the reaction mechanism simulation of Al/NiO thermite.

With the integration of data science and computing technology, machine learning has become an important means to improve the accuracy of multi-component force fields. In 2022, Smith et al. [40] innovatively trained a machine learning-enhanced ReaxFF force field (ML-ReaxFF) through the Gradient Boosting Decision Tree (GBDT) algorithm based on 10^4^ sets of high-precision DFT calculation data. This force field exhibits excellent performance when dealing with multi-component complex systems (such as the Al/CuO-SiO_2_ composite system, where SiO_2_ is an additive), with a prediction error of only 4% for the system’s interfacial diffusion coefficient, which is much lower than that of traditional empirical fitting force fields (usually with an error > 10%). This achievement not only verifies the efficiency of machine learning in force field parameter optimization but also provides a new paradigm for force field development of multi-component and multi-phase energetic systems, demonstrating the great potential of machine learning-driven force field refinement.

#### 3.2.2. Extreme Condition-Adapted Force Fields: Breakthroughs in High-Temperature, High-Pressure, and Impact Load Scenarios

The application of nAl in energetic materials is often accompanied by extreme working conditions (such as high temperature during ignition, high pressure, and impact load during explosion). However, traditional force fields are mostly developed based on normal temperature and pressure data, which are prone to problems such as inaccurate bond energy parameters and deviated reaction path prediction under extreme conditions. Therefore, force field adaptation for extreme conditions has become a research hotspot in recent years, focusing on solving the simulation accuracy problem of Al–oxidant systems under high-temperature and high-pressure conditions. Reference [41] systematically explored the oxidation kinetic mechanism of nAl under temperatures of 300–900 K and oxygen pressures of 0.13–0.26 g/cm^3^ through a ReaxFF reactive force field molecular dynamics simulation, aiming at the extreme oxidation conditions of nAl in energetic material applications. It clarified the regulatory effect of high-temperature-induced hot spot regions and void structures on oxygen diffusion barriers. By verifying and optimizing the ReaxFF force field parameters of the Al/O system, the simulation results of oxide layer growth rate and product phase distribution were qualitatively consistent with the experimental data. This successfully realized the accurate capture of oxygen diffusion paths and oxidation state transitions during the oxidation process of nAl under extreme conditions, providing important support for the refined simulation of the ignition and combustion mechanism of nAl in energetic materials.

On this basis, in 2023, Song et al. [42] systematically reviewed the development framework and optimization strategies of ReaxFF force fields, focusing on the application of global optimization methods such as multi-objective evolutionary strategy and enhanced particle swarm optimization in the calibration of force field parameters for Al–oxidant systems, aiming at the extreme working conditions such as high temperature and high pressure accompanied by the application of nAl in energetic materials. This study integrated the key technologies for a reaction simulation of Al-based energetic materials under extreme conditions, and clarified the optimization directions of traditional force fields in bond energy parameter and reaction path prediction. The summarized force field adaptation scheme significantly improved the simulation reliability of processes such as nAl oxidation under high-temperature and high-pressure conditions and the thermal decomposition of aluminum-containing composite materials, providing important method support and a theoretical reference for an accurate simulation of the combustion mechanisms of aluminum-containing energetic materials under extreme working conditions.

#### 3.2.3. Special Research: Gas–Solid Interaction Potential Function and Energy Transfer Kinetics Support

**Figure 5:**
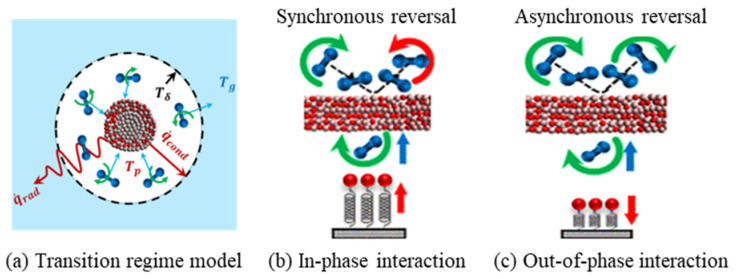
Heat transfer between the nanoparticle and gas in the non-continuum regime [43].

In addition to the aforementioned force field development for Al–oxidant systems, the interaction between nAl and gas media (such as nitrogen, oxygen) and energy transfer behavior during the combustion process are also key factors affecting its energy release efficiency. The development of related gas–solid interaction potential functions is an important supplement to simulation research in this field. In Reference [43], researchers systematically explored the energy transfer kinetics between nano-aluminum/alumina particles and nitrogen in the discontinuous medium region (where the mean free path of gas molecules is equivalent to the particle size) through the “Density Functional Theory (DFT)–Molecular Dynamics (MD)” collaborative method, as shown in Figure 5. Based on the gas–solid interaction energy data calculated by first-principles, the researchers developed an accurate gas–solid interaction potential function. This potential function can accurately describe the adsorption, collision, and energy transfer processes of nitrogen molecules on the surface of nano-aluminum/alumina, and the error between the simulated energy transfer coefficient and the molecular beam experimental results is less than 5%. This achievement not only provides a key simulation method for an in-depth understanding of the microscopic mechanism of energy transfer between nano-aluminum and gas media during the combustion process of energetic materials but also supplements the force field foundation in gas–solid interaction scenarios, further improving the force field system for a molecular dynamics simulation of nAl.

## 4. Core Value of MD Simulation in Energetic Applications of nAl

### 4.1. Surface Modification and Stability Regulation

The contradiction between the high reactivity and low storage stability of nAl is a bottleneck in its application, and surface coating modification is a key solution. Relying on atomic-level perspective and dynamic tracking capabilities, molecular dynamics (MD) simulation has realized the leap of modification from “empirical trial-and-error” to “precision design”, providing core theoretical support for scheme optimization.

#### 4.1.1. Organic Coating Mechanism: From Agglomeration Inhibition to Sintering Barrier

Organic coating layers are widely used due to their compatibility advantages, and MD simulation has revealed their stabilization mechanism. In 2021, Liu et al. [44] found through ReaxFF MD that the ethanol coating layer occupies the low-coordination sites on the Al surface, reducing the surface potential by 0.5 eV, and no agglomeration of 4 nm aluminum powder occurred at 1000 K. In 2023, their team confirmed [45] that the PTFE coating layer decomposes into Al-F clusters in the early stage of heating, forming an isolation barrier and inhibiting particle sintering at 1200 K, thus solving the problem of high-temperature failure.

#### 4.1.2. Inorganic Coating Optimization: Crystal Form Regulation and Long-Term Stability

Inorganic coating layers have high-temperature resistance, and MD simulation guides crystal form selection and material screening. In 2019, Zhang et al. [46] showed through DFT simulation that the interfacial binding energy between α-SiO_2_ and Al (−4.2 eV) is lower than that of amorphous SiO_2_ (−2.8 eV), corresponding to a 20% increase in the retention rate of active aluminum. In 2024, Wang et al. [47] screened out the AlF_3_ coating layer through MD simulation; when stored in an environment with 60% humidity for 6 months, the active retention rate still reached 85%, which was much higher than that of traditional SiO_2_ (about 60%).

#### 4.1.3. Energetic Coating Design: Balance Between Stability and Energy Performance

Energetic coatings need to balance performance, and MD simulation is a key tool. In 2017, Li et al. [48] optimized the thickness of the fluorocarbon coating to 2 nm, which increased the storage stability of the Al/CuO system by three times without the loss of combustion heat. The latest research [49] optimized the coating thickness to 3 nm through ReaxFF MD; while maintaining the combustion heat (28.5 MJ/kg), the ignition delay was reduced from 15 ms to 8 ms, realizing the coordinated optimization of performance.

#### 4.1.4. Special Research: Interface Regulation and In-Depth Verification of Coating Mechanism

**Figure 6:**
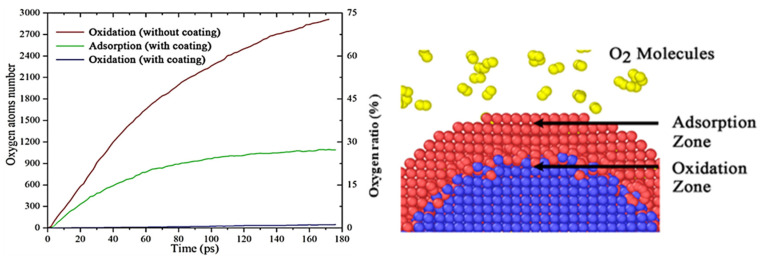
Development of adsorption and oxidation ratio in 180 ps [50].

A number of studies have combined MD with first-principles to deepen the understanding of interface mechanisms. Reference [50] compared the interfaces between three NiO surfaces and Al through AIMD simulation, and found that the diffusion energy barrier of the Octo-Top interface was only 0.28 eV (prone to pre-reaction), while the (100)-O-Top and (111)-Top interfaces had higher energy barriers (more stable), providing a basis for crystal plane selection. Reference [51] determined the optimal compatibility ratio of PDA/PTFE (10 wt%/90 wt%) through MD simulation; the composite coating had a low oxygen diffusion coefficient, and its oxidation resistance was 2.5 times higher than that of a single coating. Reference [52] revealed through ReaxFF MD that the carbon shell of carbon-coated aluminum nanoparticles (CANPs) can reduce the oxygen diffusion rate and has a “self-consumption” characteristic; after optimizing the thickness to 5 nm, the oxidation rate decreased by 80% at 800 K, as shown in Figure 6.

In summary, through revealing mechanisms, optimizing parameters, and balancing performance, MD simulation has become a core means for surface modification and stability regulation of nAl, promoting the practical application of its energetic properties.

### 4.2. Atomic-Level Analysis of Combustion Mechanism

Combustion is the core process for nAl to realize energy release, involving multi-stage elementary reactions and dynamic structural evolution. Traditional experiments are difficult to capture atomic-scale details. Relying on its ability to accurately track microscopic processes, molecular dynamics (MD) simulation has become a core tool for revealing key combustion mechanisms and correlating macroscopic properties with microscopic behaviors, providing atomic-level theoretical support for optimizing the combustion performance of nAl energetic systems.

#### 4.2.1. Resolution of Ignition Mechanism Controversy: Accurate Revelation of Dynamic Oxide Shell Rupture Process

In the ignition mechanism of nAl, the rupture mode of the oxide shell has long been a focus of controversy. In 2019, Liu’s team [1] clarified for the first time through ReaxFF MD simulation that the oxide shell does not “instantly rupture” when the Al core melts, but undergoes a three-stage dynamic process of “lattice distortion–microcrack initiation–overall rupture”. Further quantitative analysis shows that when the shell thickness decreases from 5 nm to 2 nm, the ignition delay is shortened by 60%. This result revises the traditional “instant rupture” cognition, clarifies the regulatory mechanism of shell thickness on ignition timing, and provides clear parameter basis for ignition performance optimization.

#### 4.2.2. Atomic Diffusion Kinetics: Analysis of Reaction Driving Force and Key Influencing Factors

Atomic diffusion is the core driving force for the continuous progress of combustion reactions, and MD simulation has revealed the key laws and regulatory factors of diffusion behavior. In 2013, Tang et al. [53] found through an AIMD simulation of an Al/Fe_2_O_3_ system that the diffusion coefficient of O atoms (1.2 × 10^−9^ m^2^/s) is 15 times that of Fe atoms, confirming that O atom migration is the dominant step of the reaction. In 2022, Xiong et al. [54] further verified through AIMD that the energy released by the redox reaction can maintain the continuous diffusion of Al atoms, and the formation of vacancies in the Cu layer can increase the diffusion rate by three times. These findings directly correlate the macroscopic combustion rate with the microscopic atomic transport kinetics, clarifying the driving mechanism and key regulatory factors of the diffusion process.

#### 4.2.3. Product Evolution Law: Tracking of Species Formation and Reaction Phase Transition

The type and morphology of combustion products directly affect energy release efficiency, and MD simulation has realized atomic-level tracking of product evolution. In 2017, Lin et al. [55] found through ReaxFF MD that the core product of Al/Fe_2_O_3_ combustion is Fe_2_Al clusters (bond length: 2.60–2.80 Å). In 2024, Zhu et al. [56] further revealed through simulation that when the reactant spacing is 2 nm, 14 O_2_ molecules are generated at 1900 K, prompting the reaction to shift from solid-phase dominance to a “solid–gas” multi-phase reaction. This result is completely consistent with the gaseous oxygen signal detected by mass spectrometry experiments [57], clarifying the triggering conditions of a reaction phase transition and the product evolution path.

#### 4.2.4. Special Research: Multi-Dimensional Mechanism Deepening and Application Scenario Verification

A number of targeted studies have combined MD simulation with experiments to deepen the understanding of combustion mechanisms from the dimensions of defect regulation, structural design, and medium influence, providing more detailed guidance for practical applications.

The phenomena of a “decreased combustion rate pressure index and inhibited aluminum agglomeration” observed in the experiments by Reference [58] are consistent with the conclusions of ReaxFF-MD simulation. The simulation shows that aluminum particles can change the RDX decomposition path and adjust the Al atom migration barrier, thereby reducing pressure sensitivity. At the same time, this study provides an experimental basis for an atomic-scale simulation of the entire process of “ignition–interfacial reaction–micro-explosion”. Reference [59] revealed the key role of oxygen vacancies at the Al/MoO_3_ interface through MD simulation: oxygen vacancies increase the O atom diffusion rate by two times, accelerate the formation of Al–O bonds and the breaking of Mo–O bonds, and significantly reduce the reaction energy barrier. Reference [60] studied the reaction of Al/Fe_2_O_3_ at different temperatures through AIMD simulation, and clarified that the reaction is triggered by interfacial O migration and Fe–O bond breaking. A high temperature (>1800 K) increases the reaction propagation rate by four times, and finally generates Al_2_O_3_, Fe clusters, and Al–Fe intermetallic compounds, completely analyzing the regulatory mechanism of temperature on the reaction.

Regarding the influence of interface structure and medium, Reference [61] simulated the preheating reaction of Al/CuO with porous and dense interfaces: above 600 K, pores promote O_2_ adsorption and dissociation and form an Al-rich reaction zone, increasing the reaction activity by three times; while dense interfaces tend to form an Al–Cu–O barrier layer, delaying diffusion. Reference [62] found that carbon fibers can inhibit aluminum particle sintering through the mechanism of “low-temperature physical adsorption–high-temperature Al–C bonding”, prolong their residence time in the reaction zone, and promote combustion propagation. Reference [63] simulated the reaction of the SiO_2_@Al core–shell structure and revealed that the Al_5_O intermediate product, due to its positive charge (0.32|e|), exhibits a unique diffusion behavior of “first repulsion then attraction”, which finally triggers the explosive decomposition of the system, clarifying the influence of charge effect on the reaction path. In addition, Reference [64] clarified through DFT calculations that the Al (111) surface has extremely high reactivity (barrier-free molecular dissociation), the Al_2_O_3_ surface promotes alumina epitaxial growth, while the Cu (111) surface has low reactivity. Reference [65] compared α-MnO_2_ and β-MnO_2_: α-MnO_2_ has a stable tunnel structure and reacts directly with Al to release more heat, while β-MnO_2_ destroys oxygen balance due to thermal decomposition and reduces Al utilization efficiency, providing a theoretical basis for oxidant crystal phase selection.

In summary, MD simulation realizes atomic-level analysis of the nAl combustion mechanism for the entire process of “ignition–diffusion–product evolution”, combined with multiple dimensions such as defects, structure, and medium, providing precise theoretical guidance for the performance optimization of energetic systems.

### 4.3. Interface Design of Energetic Composite Systems

In nAl-based energetic composite systems, the interfacial reaction between aluminum powder and oxidants is a core link that determines energy release efficiency, storage stability, and ignition performance. Interface bonding strength, atomic diffusion efficiency, and reaction activity directly affect the overall performance of the system. Relying on their ability to analyze interfacial behaviors at the atomic level, molecular dynamics (MD) simulation and first-principles calculation have become core tools for interface structure optimization, oxidant adaptation screening, and new system design, promoting energetic composite systems from “macroscopic ratio optimization” to “microscopic interface precise design”.

#### 4.3.1. Interface Stability Regulation: Crystal Plane Selection and Interaction Mechanism Analysis

Interface stability is the basis for long-term storage and the safe application of energetic composite systems. MD simulation and DFT calculation can accurately reveal the regulatory mechanism of crystal plane characteristics and its impact on interface interactions. In 2012, Lanthony et al. [66] found through DFT calculation that the adsorption behavior of Al atoms on different CuO crystal planes is significantly different: the adsorption energy on the CuO (11-1) surface is -5.08 eV, which is significantly lower than that on the (001) surface (−3.2 eV). The heat released by the stronger adsorption can overcome the 0.6 eV penetration energy barrier of Al atoms, providing a parameter basis for the balance between interface stability and reaction initiation. In 2022, Xue et al. [10] further studied the interfacial interaction between Al and Fe_2_O_3_ through the DFT and confirmed that the Fe_2_O_3_ (104) surface with exposed O atoms forms the maximum interfacial adhesion work (3.8 J/m^2^) with Al. The differential charge density map shows significant charge accumulation in the interface region, indicating that this crystal plane can improve interface bonding stability through strong electronic interaction, providing clear theoretical guidance for oxidant crystal plane selection.

#### 4.3.2. Interface Reaction Kinetics Regulation: Key Factors and Optimization Strategies

The interfacial reaction kinetics of energetic composite systems is jointly regulated by multiple factors such as interface bonding mode, atomic diffusion barrier, and defect distribution. MD simulation can quantitatively analyze the influence of each factor and provide targeted optimization strategies. For example, Reference [67] found through DFT calculations that the (310) crystal plane of MnO_2_ has the smallest oxygen vacancy formation energy (1.09 eV), which can reduce the O atom diffusion barrier at the interface by 30%, thereby increasing the interfacial reaction rate. Reference [61] showed that the porous interface of Al/CuO can reduce the atomic diffusion distance by 50% compared to the dense interface, significantly accelerating the interfacial reaction. In addition, the introduction of appropriate defects (such as oxygen vacancies, dislocations) at the interface can also reduce the reaction activation energy: Reference [59] confirmed that oxygen vacancies at the Al/MoO_3_ interface can reduce the reaction activation energy from 1.2 eV to 0.5 eV, significantly improving the reaction kinetics. These studies show that regulating the interface structure and defect state through MD simulation-guided design is an effective way to optimize the interfacial reaction kinetics of energetic composite systems.

#### 4.3.3. Special Research: Multi-Scenario Interface Mechanism Deepening and Design Strategies

A number of special studies have combined simulation with experiments to deepen the understanding of interface design from the dimensions of mechanical properties, pre-reaction mechanisms, and crystal plane engineering, providing detailed strategies for complex scenario applications. Reference [68] focuses on the interface mechanics and stability of an Al/NiO system. Through first-principles tensile testing and AIMD simulation, it compares the behaviors of two interface configurations, (100)-Top and (111)-Top, under the “stress-temperature” coupling field. It is found that the (100)-Top interface fractures near the NiO layer (due to the easy dissociation of Ni–O bonds), while the (111)-Top interface fractures in the Al layer (weak Al–Al bond bonding); moreover, the coupling of pre-stress and a high temperature induces the formation of an interfacial amorphous oxide layer, accelerating element diffusion. This study provides an atomic-level design strategy for regulating the mechanical integrity and storage stability of the system through crystal plane orientation selection. Reference [69] reveals the pre-ignition reaction mechanism between PVDF and the alumina shell on the n-Al surface through AIMD: F atoms at the end of the PVDF chain first adsorb on the alumina surface to form a C–F–Al structure, and then F dissociates and forms stable Al–F bonds with Al, destroying the integrity of the oxide shell and opening channels for subsequent Al-O reactions. This discovery explains the “ignition promotion” effect of fluoropolymers on aluminum-based systems. Reference [67] compares the interactions of different MnO_2_ crystal planes through DFT and finds that the (310) crystal plane has the lowest surface energy (1.12 J/m^2^) and the smallest oxygen vacancy formation energy (1.09 eV), which can significantly promote oxygen activation and migration, increasing the heat release of the thermite reaction by 25%, providing a basis for oxidant crystal plane engineering.

In summary, through analyzing interface interaction mechanisms, screening suitable components, and optimizing structural parameters, MD simulation and first-principles calculation have become core technical supports for the interface design of energetic composite systems, promoting the precise breakthrough of system performance towards the direction of “high energy, high stability, and scenarioization”.

## 5. Research Challenges and Future Outlook

### 5.1. Current Core Challenges

Although significant progress has been made in the microscopic simulation and theoretical research of nAl energetic systems, there are still key bottlenecks in simulation condition coverage, multi-scale correlation, and experimental verification, which restrict the transformation from atomic-level mechanisms to engineering applications. These challenges are interrelated but distinct: the first challenge lies in the lack of mature formalisms and models to introduce multiple loads (e.g., impact, laser, extrusion) simultaneously in MD simulations (i.e., “lack of multi-load simulation”); the second is insufficient multi-scale coupling. The interconnection between them is that actual engineering scenarios involve complex multi-loads that span atomic, mesoscopic, and macroscopic scales, and the absence of accurate multi-load simulation models makes it difficult to obtain reliable atomic/mesoscopic reaction data for multi-scale coupling, thereby increasing the difficulty of establishing a direct link between atomic-level mechanisms and macroscopic performance.

#### 5.1.1. Lack of Simulation Under Multi-Load Coupling Conditions

The existing simulation studies are mostly confined to single isothermal or heating conditions [4]. A typical example is the research on Al/SiO_2_ thermite systems by Zhang et al. [12]: They employed molecular dynamics (MD) simulation coupled with reactive force field potential functions to investigate the thermite reaction of Al/SiO_2_-layered nanostructures, conducting the entire simulation under adiabatic conditions and treating thermal load as the sole driving factor. Specifically, they designed six initial temperature settings (600 K, 700 K, 800 K, 900 K, 1000 K, 1100 K) to explore how the system behaves under different thermal conditions—yet throughout the process, they only regulated and analyzed temperature-related parameters, with no introduction of other external loads such as impact, extrusion, or laser. This reflects a common limitation in current studies: an over-reliance on single thermal load settings.

However, in real-world energetic application scenarios, thermite reactions are often induced by the coupling of multiple loads, including impact-explosive driving, laser ignition, and extrusion-temperature effects. Different loads give rise to unique interfacial dynamic behaviors: for instance, impact leads to frictional heating, while laser triggers a local rapid temperature rise via photothermal conversion. At present, molecular dynamics (MD) simulation models for such complex load conditions remain immature; in particular, there is a lack of unified formalisms to integrate multiple physical loads (mechanical, thermal, optical) into a single simulation framework. The singularity of the existing simulation setups—with most focus on temperature-driven processes under NVT/NVE ensembles and a lack coverage of multi-load coupling—makes it impossible to accurately predict the reaction kinetics and energy release characteristics of nAl in complex environments [70]. Notably, the Multi-Scale Shock Technique (MSST) is an example of a technique designed to handle specific multi-physics scenarios (e.g., shock wave propagation coupled with thermal effects), but it is not a universal solution for all multi-load combinations (e.g., laser–thermal–mechanical coupling) and has limited applicability in simulating the full range of complex load conditions encountered in nAl-based energetic material applications.

#### 5.1.2. Lack of Explicit Mesoscale Description

There is a difference of 2–3 orders of magnitude between the spatiotemporal scale of MD simulation (space: 1–10 nm, time: 1–100 ns) and macroscopic experiments (space: 20–100 nm, time: 1–100ms), resulting in atomic-level simulation results that cannot be directly correlated with key engineering parameters such as combustion wave velocity and propellant specific impulse. The mesoscopic scale (10–100 nm) is a key link connecting the atomic scale and macroscopic scale, involving core processes such as particle agglomeration, interface evolution, and local hot spot formation. However, the existing studies lack an explicit mesoscale description and corresponding simulation models to characterize the structural evolution and reaction kinetics at this scale. For example, the correlation between atomic-level mechanisms (e.g., atomic diffusion, bond breaking) and mesoscale phenomena (e.g., agglomerate formation, heterogeneous reaction front propagation) has not been systematically established. Although Zhou et al. [71] constructed an MD-Computational Fluid Dynamics (CFD) multi-scale coupling model, attempting to obtain reaction kinetic parameters (such as reaction rate constant) through MD and input them into CFD for macroscopic prediction, the mesoscale is omitted, leading to a prediction error of macroscopic parameters that still exceeds 15%; in addition, the existing coupling models do not consider the influence of mesoscale agglomerate formation on the reaction, which further limits their engineering application value.

#### 5.1.3. Difficulties in Experiment-Simulation Quantitative Verification

The mismatch between the spatiotemporal resolution of experimental characterization technology and simulation makes it difficult to establish a quantitative verification system. On the one hand, the time resolution of in situ characterization technologies (such as in situ TEM and femtosecond laser spectroscopy) is mostly at the picosecond (ps) level, while the time resolution of ab initio molecular dynamics (AIMD) simulation can reach the femtosecond (fs) level, making it impossible to directly compare transient processes such as O atom migration and chemical bond breaking [72]. On the other hand, there is a lack of corresponding experimental measurement methods for microscopic parameters obtained from simulations, such as the atomic diffusion coefficient and interfacial charge transfer amount.

### 5.2. Future Development Directions

To address the above challenges, future research needs to make breakthroughs in three aspects: multi-field coupling simulation, intelligent force field development, and experiment-simulation closed-loop construction, promoting the in-depth integration of theoretical research and the engineering application of nAl energetic systems.

#### 5.2.1. Development of Multi-Field Coupling Simulation Technology

For complex loads such as impact and laser, it is necessary to establish a “mechanical–thermal–optical” multi-field coupled MD model. Specifically, the Multi-Scale Shock Technique (MSST) should be introduced to describe the propagation and energy dissipation of shock waves in nAl-based energetic systems [73], while the two-temperature model (separating electron temperature from lattice temperature) can be integrated to simulate photothermal conversion and energy transfer processes under laser loading [74]. Ge et al. [75] has successfully simulated the shock response of RDX explosives through this idea, providing a technical reference for the multi-field coupling simulation of energetic systems. At the same time, it is necessary to construct a four-level coupling platform of “DFT–AIMD–ReaxFF MD–mesoscopic/CFD” that uses DFT to provide electronic structure parameters, AIMD to reveal the initial reaction mechanism, and ReaxFF MD to obtain large-system reaction kinetic data (such as reaction rate, product distribution), and finally input these atomic-level data into mesoscopic models (such as phase-field model) and CFD to realize quantitative prediction from atomic behavior and macroscopic parameters such as combustion performance [75].

#### 5.2.2. Breakthroughs in Intelligent Force Fields and Efficient Computing

Relying on machine learning and high-throughput computing, construct a force field database covering 10^5^ levels of experimental data and DFT calculation data, optimize the force field parameters through algorithms such as gradient boosting and neural networks, and control the simulation error of multi-component energetic systems (such as Al/oxidant/additive) within 5%. The ML-ReaxFF force field developed by Smith et al. [40] has shown this potential in the Al/CuO-SiO_2_ system, and it is necessary to further expand to complex nAl-based composite systems involving energetic ionic salts or fluoropolymers as oxidants/additives in the future. In terms of computing efficiency, GPU parallel acceleration and domain decomposition algorithms are adopted to expand the simulation system scale of ReaxFF MD from 10^4^ atoms to 10^6^ atoms, and the computing efficiency is increased by more than 20 times [76], realizing the dynamic evolution simulation of micron-level energetic systems (such as multi-particle agglomerates) and filling the gap in mesoscopic scale simulation.

#### 5.2.3. Construction of Experiment-Simulation Closed-Loop System

Develop in situ characterization technology with a high spatiotemporal resolution: combine a femtosecond pulse laser with in situ TEM to increase the time resolution to 100 fs, realizing a direct comparison with atomic trajectories of AIMD simulation [33]; use neutron scattering technology (such as pulsed neutron source) to measure atomic diffusion coefficients, providing quantitative verification data for MD simulation [53].

Establish a digital twin model of an nAl energetic system by building an intelligent design platform for nano-energetic materials, and using the platform’s closed-loop process of “simulation design–experimental verification–force field correction” to iteratively optimize simulation parameters and experimental schemes, which can greatly shorten the R&D cycle of new energetic systems. For example, Li et al. [77] predicted the combustion performance of the Al/PTFE system through a reactive molecular dynamics (RMD) simulation, and then verified and corrected the force field parameters through laser ignition experiments, which provides a successful example for the construction of such closed-loop systems.

## 6. Conclusions

The application of nAl in energetic materials has advanced from traditional macroscopic performance optimization to a new stage of microscopic regulation based on atomic-level mechanisms. The core–shell structural characteristic of nAl, i.e., “Al core–Al_2_O_3_ shell”, determines its oxidation activity and storage stability; while the interfacial reaction behavior between aluminum powder and oxidants dominates the ignition characteristics and energy release efficiency of energetic composite systems. Molecular dynamics simulation technologies, especially the synergistic application of ReaxFF MD and AIMD, have successfully revealed core reaction mechanisms such as diffusion-oxidation and metal–oxygen inversion, and realized targeted optimizations including coating structure design and oxidant adaptation screening, providing crucial theoretical support for the performance optimization of energetic systems.

Although significant breakthroughs have been achieved in recent three years in aspects such as multi-component force field development and new composite system design, core challenges still remain, including the lack of multi-load simulations, insufficient multi-scale coupling, and difficulties in experiment-simulation quantitative verification. In the future, the precise design and performance prediction of nAl energetic systems are expected to be realized by developing multi-field coupling simulation technologies, intelligent force fields and efficient computing methods, and constructing an experiment-simulation closed-loop system. In particular, the in-depth integration of machine learning and molecular dynamics simulation will promote the transition of the R&D of energetic materials into a new stage of efficient iteration featuring “theoretical prediction–experimental verification”, providing solid theoretical and technical support for the energetic materials.

## Figures and Tables

**Table 1:** Applicable conditions of three oxidation mechanisms of nAl.

Oxidation Mechanism	Key Trigger Conditions	Dominant Particle Size Range	Typical Application Scenarios
Diffusion-oxidation	Heating rate ≤ 10^5^ K/s, normal pressure	30–100 nm	Slow ignition, long-term storage oxidation
Melting-dispersion	Heating rate ≥ 10^6^ K/s, pressure ≥ 2.3 MPa	10–50 nm	Explosion, pulse heating, high-energy ignition
Ion diffusion	Temperature 800–2000 K, intrinsic electric field in Al_2_O_3_ shell	20–80 nm	Electrochemical reaction, medium-temperature rapid oxidation

**Table 2:** Initial reaction temperatures of Al/NiO thermite at different reaction stages [32].

Simulation/Experimental Conditions	Initial Ignition Temperature/K	Primary Exotherm Initiation Temperature/K	Secondary Exotherm Initiation Temperature/K
10 → 1500 K Heating Simulation (^16^O)	522	729	909
10 → 1500 K Isothermal Simulation (^18^O)	730	904	1142
Experiment 1 (DSC)	–	742	918
Experiment 2 (TG-DTA)	–	774	918
Experiment 3 (Laser Ignition)	–	733	839
Experiment 4 (High-Pressure DSC)	–	673	803

## Data Availability

The original contributions presented in this study are included in the article. Further inquiries can be directed to the corresponding authors.
